# Supplementary material for: Snapshot of the Probiotic Potential of Kluveromyces marxianus DMKU-1042 Using a Comparative Probiogenomics Approach
Source: Foods. 2023 Nov 30;12(23):4329. doi: 10.3390/foods12234329 (PMC10706368; doi:10.3390/foods12234329)
Supplement: Supplementary file 1 [file foods-12-04329-s001.zip › foods-2707500-supplementary.pdf]

Supplementary Table S1: Source information, and general genome features of *Kluyveromyces marxianus* strains

WGS	Strains	BioSample	No of contigs	Contig N50(Mb)	Contig L50	Isolation source	Region	GC Content (%)	Submitter	Year	Sequencing platform
**BBIL00000000**	DMB1	SAMD00017758	36	1.4	4	Sugarcane	Japan	40.09	National Institute of Advanced Industrial Science and Technology	2014	PacBio RS II
**LDJA00000000**	IIPE453	SAMN01103505	707	0.0353	93	Sugar mill waste	India	40.17	CSIR-Indian Institute of Petroleum	2012	Illumina NextSeq
**LDJA00000000**	UFS-Y2791	SAMN04590183	1088	0.0213	159	Agave plant	South Africa	40.04	University of the Free State	2007	Illumina HiSeq
**CP015054**	FIM1	SAMN04601176	8	1.4	4	Yogurt	China	40.16	Fudan university	2016	Illumina Hiseq 2000
**CP023456**	NRRL Y-6860	SAMN06013971	9	1.4	4	Banana alcoholic beverage	Ecuador	40.20	J. Craig Venter Institute	1971	PacBio
**CP015054**	LHW-O	SAMN06765481	480	0.0401	88	Onion	South Korea	40.15	World Institute of Kimchi	2016	Illumina MiSeq
**CP015054**	L03	SAMN12504567	262	0.0694	52	Dairy	Ireland	40.13	Wolfe Lab, Conway Institute, University College Dublin	2019	Illumina HiSeq
**CP015054**	Olga-2	SAMN08581128	1154	0.0182	74	Kefir	Germany	40.19	SysMilk	2016	Illumina HiSeq
**PUHV00000000**	Olga-1	SAMN08581126	973	0.0201	147	kefir	Germany	40.13	Chalmers University of Technology	2016	Illumina HiSeq
**JAKGCN000000000**	KM3	SAMN24619240	20	1.0	4	Tibetan kefir	China	40.21	Technology Center of Bright Dairy & Food Co., Ltd, China	2012	Illumina HiSeq; PacBio

**AP014599**	NBRC1777	SAMD00017339	8	1.4	4	-	Japan	40.12	Organization of Advanced Science and Technology, Kobe University	2014	PacBio RS II; Ion Torrent
**CP067318**	CBS6556	SAMN17225432	8	1.4	4	Fermented maize dough	Mexico	40.21	Delft University of Technology	1971	Oxford Nanopore MinION
**JAIRBU000000000**	SLP1	SAMN18296546	38	0.3689	9	fermentation tanks	Mexico	39.5	Centro de Investigation y AsistenciaenTecnologia y Diseno del Estado de Jalisco AC	2009/2010	PacBio RSII
**AKFM00000000**	KCTC1755	SAMN02981417	111	1.2	4	Pozol	Mexico	40.14	Korea Research Institute of Bioscience and Biotechnology	2014	Illumina GAIIx
**AP012213**	DMKU3-1042_GCF	SAMD00067077	8	1.4	4	Budding yeast	Japan	40.12	Yamaguchi University	2014	Sanger ABI3730

Supplementary Table S2. Amino acids and their putative functions detected in the *K. marxianus* DMKU3-1042 isolates.

Amino acids	Putative functions
**Lysine, threonine, methionine and cysteine**	homoserine kinase<br>homoserine dehydrogenase<br>aspartokinase<br>Aspartate-semialdehyde dehydrogenase<br>threonine synthase<br>S-methyl-5-thioribose-1-phosphate isomerase<br>homocysteine S-methyltransferase<br>2,3-diketo-5-methylthiopentyl-1-phosphate enolase-phosphatase<br>Methylthioribulose-1-phosphate dehydratase<br>5'-methylthioadenosine phosphorylase<br>1,2-dihydroxy-3-keto-5-methylthiopentene dioxygenase
**Histidine metabolism**	Imidazoleglycerol-phosphate dehydratase<br>Histidinol-phosphate aminotransferase<br>Imidazole glycerol phosphate synthase<br>Adenylosuccinate synthetase<br>Phosphoribosylformimino-5-aminoimidazole carboxamide ribotide isomerase
**Aromatic amino acids and derivatives**	Prephenate dehydratase<br>Chorismate mutase III<br>Prephenate and/or arogenate dehydrogenase<br>Phosphoribosylanthranilate isomerase

	Tryptophan synthase beta chain<br><br>Anthranilate synthase
**Proline and 4-hydrxyproline**	Gamma-glutamyl phosphate reductase<br><br>Glutamate-5-kinase<br><br>pyrroline-5-carboxylate reductase<br><br>NADP-specific glutamate dehydrogenase<br><br>delta-1-pyrroline-5-carboxylate dehydrogenase<br><br>Argininosuccinate synthase<br><br>argininosuccinate lyase<br><br>ornithine aminotransferase<br><br>N-acetylornithine aminotransferase<br><br>Arginase
**Alanine, serine and glycine**	phosphoserine aminotransferase<br><br>aminomethyltransferase<br><br>glycine dehydrogenase<br><br>glycine cleavage system H protein
**Glutamine, glutamate, aspartate, asparagine, ammonia**	Aspartokinase<br><br>homoserine dehydrogenase<br><br>Glutamine synthetase type II<br><br>NAD-specific glutamate dehydrogenase<br><br>NADP-specific glutamate dehydrogenase<br><br>Aspartate-semialdehyde dehydrogenase
**Arginine; urea cycle, creatine and polyamines**	N-acetylglutamate kinase<br><br>argininosuccinate lyase<br><br>ornithine carbamoyltransferase<br><br>Glutamate N- acetyltransferase<br><br>Spermidine synthase<br><br>Arginase<br><br>Argininosuccinate synthase

	Allophanate hydrolase
